# Perturbed CD8^+^ T cell TIGIT/CD226/PVR axis despite early initiation of antiretroviral treatment in HIV infected individuals

**DOI:** 10.1038/srep40354

**Published:** 2017-01-13

**Authors:** Johanna Tauriainen, Lydia Scharf, Juliet Frederiksen, Ali Naji, Hans-Gustaf Ljunggren, Anders Sönnerborg, Ole Lund, Gustavo Reyes-Terán, Frederick M. Hecht, Steven G. Deeks, Michael R. Betts, Marcus Buggert, Annika C. Karlsson

**Affiliations:** 1 Division of Clinical Microbiology, Department of Laboratory Medicine, Karolinska Institutet, Karolinska University Hospital Huddinge, Stockholm, Sweden; 2 Center for Biological Sequence Analysis, Department of Systems Biology, Technical University of Denmark, Lyngby, Denmark; 3 Division of Transplantation, Department of Surgery, Perelman School of Medicine, University of Pennsylvania, Philadelphia, PA, United States of America; 4 Center for Infectious Medicine, Department of Medicine, Karolinska Institutet, Karolinska University Hospital, Stockholm, Sweden; 5 Unit of Infectious Diseases, Department of Medicine Huddinge, Karolinska Institutet, Karolinska University Hospital Huddinge, Stockholm, Sweden; 6 Centre for Infectious Diseases Research, National Institute of Respiratory Diseases, Mexico City, Mexico; 7 Department of Medicine, University of California, San Francisco Positive Health Program, San Francisco General Hospital, San Francisco, CA, United States of America; 8 Department of Microbiology, Perelman School of Medicine, University of Pennsylvania, Philadelphia, PA, United States of America

## Abstract

HIV-specific CD8^+^ T cells demonstrate an exhausted phenotype associated with increased expression of inhibitory receptors, decreased functional capacity, and a skewed transcriptional profile, which are only partially restored by antiretroviral treatment (ART). Expression levels of the inhibitory receptor, T cell immunoglobulin and ITIM domain (TIGIT), the co-stimulatory receptor CD226 and their ligand PVR are altered in viral infections and cancer. However, the extent to which the TIGIT/CD226/PVR-axis is affected by HIV-infection has not been characterized. Here, we report that TIGIT expression increased over time despite early initiation of ART. HIV-specific CD8^+^ T cells were almost exclusively TIGIT^+^, had an inverse expression of the transcription factors T-bet and Eomes and co-expressed PD-1, CD160 and 2B4. HIV-specific TIGIT^hi^ cells were negatively correlated with polyfunctionality and displayed a diminished expression of CD226. Furthermore, expression of PVR was increased on CD4^+^ T cells, especially T follicular helper (Tfh) cells, in HIV-infected lymph nodes. These results depict a skewing of the TIGIT/CD226 axis from CD226 co-stimulation towards TIGIT-mediated inhibition of CD8^+^ T cells, despite early ART. These findings highlight the importance of the TIGIT/CD226/PVR axis as an immune checkpoint barrier that could hinder future “cure” strategies requiring potent HIV-specific CD8^+^ T cells.

During chronic HIV-1 infection CD8^+^ T cells gradually lose their cytotoxic function, production of antiviral cytokines and their capacity to proliferate^1^,^2^,^3^,^4^ (reviewed in ref. 5). In addition, these cells accumulate cell surface markers associated with immune dysfunction, *e.g.*, inhibitory receptors such as PD-1, CD160, 2B4, TIM-3, and LAG-3^6^,^7^,^8^,^9^,^10^,^11^,^12^,^13^,^14^. This pattern persists even after years of successful antiretroviral treatment (ART)^9^,^10^. The function of the CD8^+^ T cells is partly regulated by the T-box transcription factors T-bet and Eomesodermin (Eomes) that control effector function and memory differentiation^15^,^16^. HIV-specific cells display a specific pattern of T-box transcriptions factors (T-bet^dim^Eomes^hi^) that is coupled to the increased expression of inhibitory receptors^9^. Together, these processes result in an exhausted population of HIV-specific CD8^+^ T cells that accumulates over time and impairs optimal control of the infection, even after successful ART.

Recently, T cell immunoglobulin and immunoreceptor tyrosine-based inhibitory motif (ITIM) domain (TIGIT) was identified as an inhibitory receptor expressed by human T cells and natural killer (NK) cells^17^,^18^,^19^. TIGIT inhibits T cell and NK cell function through several mechanisms including signaling through its ITIM domain^18^ induction of immunoregulatory dendritic cells^19^ and by blocking cytotoxicity^18^,^20^. Furthermore, TIGIT is associated with a downregulation of the co-stimulatory receptor CD226 and molecules involved in TCR signaling^17^,^21^.

TIGIT expression is increased on CD8^+^ T cells in HIV-infection and in cancer^21^,^22^ and co-blockade of TIGIT and the PD-1/PD-L1 pathway was recently shown to in part restore the proliferative capacity and/or cytokine production of CD8^+^ T cells in some HIV-infected subjects^23^ and cancer patients^21^,^22^ *in vitro*, making these pathways promising candidates for restoration of CD8^+^ T cell responses. Differences in response to inhibitory receptor blockade treatment have also been shown in murine studies and in human T cells from HIV-infected subjects, where not all individuals responded equally to treatment^24^,^25^. Therefore, the potential usefulness of HIV-specific CD8^+^ T cells to eliminate target cells in immune based curative strategies requires further investigation with a particular emphasis of the T cell exhaustion process, including the role of TIGIT. Recently, CD4^+^ T cells expressing TIGIT, alone or together with PD-1 and/or LAG-3 were shown to be enriched for persistent HIV during ART, suggesting that inhibitory receptor blockade treatment may also have an effect on latently infected CD4^+^ T cells^26^.

TIGIT shares its ligands, poliovirus receptor (PVR, CD155) and Nectin-2 (PVRL2, CD112), with the co-stimulatory receptor CD226 (also known as DNAM-1)^19^. Expression of CD226 is downregulated during chronic HIV infection^27^. Additionally, TIGIT is able to suppress CD226 expression by directly hindering the dimerization of CD226^21^. Hence, TIGIT and CD226 are emerging as an inhibitory/stimulatory receptor pair of importance for CD8^+^ T cell function, similar to CTLA-4 and CD28 (reviewed in ref. 28). However, the link between TIGIT and CD226 in the context of HIV infection has not been evaluated, nor has the relationship of TIGIT to the inhibitory receptors CD160 and 2B4 or transcription factors involved in T cell differentiation. Additionally, expression of the TIGIT/CD226 ligand PVR is upregulated on cancer cells^29^,^30^ and HIV proteins are able to regulate PVR expression on CD4^+^ T cells infected with HIV *in vitro*^31^,^32^. However, the expression of PVR on *ex vivo* CD4^+^ T cells from HIV-infected subjects has not been described.

Here, we show that TIGIT and CD226 are differentially expressed on HIV-specific CD8^+^ T cells. Strikingly, elevation of TIGIT expression levels was detected in longitudinal samples from HIV infected subjects treated from early infection. Increased expression of TIGIT during HIV-1 infection was coupled to a transitional T-bet^dim^Eomes^hi^ transcriptional phenotype and decreased functional capacity of HIV-specific CD8^+^ T cells. Furthermore, increased expression of the TIGIT/CD226 ligand PVR on CD4^+^ T cells in HIV-infected subjects was observed, especially on T follicular helper cells (Tfh), which are a major compartment of productive and latent HIV-infection^33^,^34^. Overall, these results highlight the important role of the TIGIT/CD226/PVR axis in T cell exhaustion and control of HIV-infection.

## Materials and Methods

### Human subjects and ethical statement

Blood samples from 30 treatment-naïve HIV-positive subjects, 20 HIV-positive subjects on long-term ART and 26 HIV-negative healthy controls were collected at the HIV clinics at Karolinska University Hospital in Huddinge and Venhälsan at Stockholm South General Hospital (Table 1). Cryopreserved peripheral blood mononuclear cells (PBMCs) from subjects with acute HIV-infection (n = 12) and elite controller subjects (n = 14) were acquired from the OPTIONS^35^ and SCOPE^36^ cohorts, respectively, at University of California San Francisco, USA (Table 1). Samples from subjects with acute HIV-infection were collected within 24–43 days (median 26.5) after the estimated infection date and all subjects initiated ART during acute HIV-infection. Of the 12 individuals with acute HIV-infection, 10 were followed longitudinally with samples collected at baseline (median 24 days), 6 months post-ART initiation (median 5.5 months) and 1.5–12 years (median 3.2 years) after the estimated infection date. Lymph nodes and matched blood samples were collected from 8 HIV-positive individuals at the Centre for Infectious Diseases Research, National Institute of Respiratory Diseases, Mexico City, Mexico. As controls, lymph nodes and blood were collected from HIV-negative subjects at the Division of Transplantation, Department of Surgery, Perelman School of Medicine, University of Pennsylvania, Philadelphia, USA. Lymph node mononuclear cells (LNMCs) were isolated through mechanical disruption of lymph nodes, either manually or according to the manufacturer’s instructions for the gentleMACS tissue dissociator (Miltenyi Biotec).

The Regional Ethical Council, Stockholm, Sweden (2014/842-32, 2009/1592-32) approved the study. The institutional review board of the University of California, San Francisco, California, USA approved the use of samples from OPTIONS and SCOPE. The Institutional Review Board at the University of Pennsylvania, Philadelphia, USA, approved collection of the lymph node and blood samples from HIV-negative subjects and the Institutional Review Board at the Centre for Infectious Diseases Research, Mexico City, Mexico approved collection of blood and lymph node samples from HIV infected subjects. Written informed consent was documented from all study subjects in accordance with the Declaration of Helsinki. All methods utilized for this study were performed in accordance with the relevant guidelines and regulations.

### Antibody reagents

The following antibodies were used for extra- or intracellular staining of PBMCs; CCR7 PECy7(Clone 3D12), CD3 V500 (Clone UCHT1), CD3 APC-H7 (Clone SK7) CD4 A700 (Clone RPA-T4), CD11c BV711 (Clone B-ly6), CD14 V500 (Clone M5E2), CD16 APC-H7 (Clone 3G8), CD19 V500 (Clone HIB19), CD19 BV711 (Clone SJ25C1), CD21 PECF594 (Clone B-ly4), CD35 BV421 (Clone E11), CD38 BV711 (Clone HIT2), CD45RO BV650 (Clone UCHL1), CD56 AF488 (Clone B159), CD107a PE-CF594 (Clone H4A3), CD123 PE-Cy7 (Clone 7G3), CD160 A488 (Clone BY55), CD226 FITC or BV711 (Clone DX11), GrzB A700 (Clone GB11), HLA-DR BV605 (Clone G46-6), IFNg A700 (Clone B27), TNF PE-Cy7 or PeCF594 (Clone MAb11) were purchased from BD Bioscience. CCR7 APC-Cy7 (Clone G043H7), CD3 BV570 (Clone UCHT1), CD4 BV785 (Clone OKT4), CD8 BV605 (Clone RPA-T8), CD14 BV570 (Clone M5E2), CD19 BV785 (Clone HIB19), CD27 BV785 (Clone O323), IFN-γ APC-Cy7 (Clone 4 S.B3), PD-1 PE-Cy7 or BV421 or BV785 (Clone EH12.2H7), Perforin BV421 (Clone B-D48) PVRL2 APC (Clone TX31) and T-bet BV605 or T-bet PE (Clone 4B10) were purchased from Biolegend. CD8 PE-Cy5.5 (Clone RPA-T8), CD38 APC (Clone HIT2), Eomes eFluor660 (Clone WD1928), CXCR5 PEeFluor610 (Clone MU5UBEE), PVR PE (Clone 2H7CD155), TIGIT PE, PE-Cy7 or PerCPeFluorF710 (Clone MBSA43) were purchased from eBioscience. CD8 Qdot565 (Clone 3B5), Granzyme B PE-Cy5.5 (Clone GB11) and CD4 PE-Cy5.5 (Clone S3.5) were purchased from LifeTechnologies/Invitrogen. 2B4 PE-Cy5 (Clone C1.7) and CD45RO ECD (Clone UCHL1) were purchased from Beckman Coulter. LIVE/DEAD® Fixable Aqua Dead Cell Stain Kit from Life Technologies was used to stain for dead cells. For tetramer analysis, the HLA-A*0201 restricted tetramers HIV Gag SLYNTVATL (SL9) conjugated to PE and CMV pp65 NLVPMVATV (NV9) conjugated to PE (MBL) were used.

### Cell preparation and antigens

Density gradient centrifugation with Hypaque-Ficoll (GE Healthcare), was used to isolate PBMCs from whole blood. The isolated cells were cryopreserved in FBS (LifeTechnologies) containing 10% DMSO. Cryopreserved PBMCs were thawed and washed twice in R10 media (RPMI-1640 Medium AQ Media (Sigma Aldricht) containing 10% FBS, 50 IU/mL penicillin, 50 μg/mL streptomycin and 10 mM HEPES (LifeTechnologies)). Cells were rested for 6 hours at 37 °C. Peptide pools (15-mers overlapping by 11 amino acids were used at a concentration of 1 μg/mL to detect HIV Gag p55 (JPT Technologies) and HCMV pp65 (JPT Technologies) specific CD8^+^ T cell responses.

### Stimulation and extra- and intracellular staining of cells for flow cytometry

Stimulation and intracellular staining were performed as described previously^9^. Briefly, cells (1–1.5 × 10^6^/well) were stimulated with overlapping HIV Gag-p55 and HCMV pp65 peptides or medium alone (negative controls) in the presence of anti-CD107a antibody, monensin (0.7 mg/mL, BD Bioscience) and Brefeldin A (5 μg/mL, BD Bioscience). After 10 hours cells were washed with PBS:EDTA and stained with LIVE/DEAD Aqua amine dye solution containing the extracellular antibodies. The cells were incubated for 30 minutes at room temperature; alternatively, for staining with anti-CCR7 antibodies, cells were incubated at 37 °C for 10 minutes followed by 20 minutes in room temperature. For extracellular staining only, cells were subsequently washed and fixed with PBS + 1% paraformaldehyde. For the intracellular staining, cells were fixated and permeabilized using the FoxP3 transcription factor buffer kit (eBioscience) according to the manufacturer’s protocol, followed by staining with intracellular markers and incubation for 60 minutes at room temperature. The cells were washed and resuspended in PBS + 1% paraformaldehyde.

### Flow cytometry analysis

PBMCs and LMNCs were analyzed on a modified 4-laser LSR Fortessa (BD Biosciences). Antibody capture beads (BD Bioscience) were used for single-stain compensation controls. Flow cytometry data were analyzed with FlowJo 8.8.7 (Treestar) and when applicable, fluorescence minus one (FMO) gating strategies were used to set the manual gates^9^,^37^,^38^. A virus-specific response was considered positive when ≥0.05% of the cells produced IFN-γ after background reduction and if the response was larger than twice the negative background signal^9^,^37^,^38^.

### Statistics

The matlab based tool, CYT, was used to generate and visualize the viSNE maps^39^. This analysis was performed to enable the visualization of complex expression patterns of cell surface markers on CD8^+^ T cell subsets. Comparisons between two groups of individuals were performed with the Mann-Whitney U test, and the Wilcoxon matched-pairs rank test was used to compare paired samples. The non-parametric Spearman rank test was used for correlation analysis. One-way ANOVA with repeated measures, followed by Kruskal-Wallis non-parametric Dunn’s multiple comparison tests were used to compare three or more groups simultaneously. The statistical tests were performed in GraphPad Prism 5.0. Permutation tests were performed in SPICE version 5.3003^40^. In the figures statistically significant values are indicated by one to three stars; *P < 0.05, **P < 0.01 and ***P < 0.001).

## Results

### TIGIT is expressed on effector/memory CD8^+^ T cell populations in healthy and HIV-infected individuals

**Figure 1:**
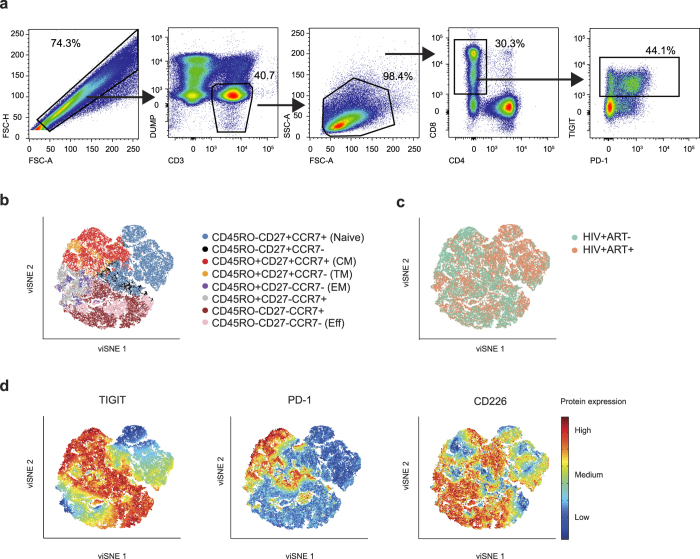
Expression patterns of TIGIT, PD-1 and CD226 on naïve/memory CD8^+^ T cells. (**a**) Gating strategy for TIGIT expression on CD8^+^ T cells in a representative subject. (**b**) viSNE map of naïve/memory phenotype distribution (based on CD45RO, CCR7 and CD27) in HIV+ subjects. Colors depict memory phenotypes. (**c**) Distribution of cells based on treatment status, HIV+ART− (green, n = 6) and HIV+ART+ (orange, n = 6) based on memory populations shown in (**b**). (**d**) Distribution of TIGIT, PD-1 and CD226 based on memory populations shown in (**b**). Colors depict intensity of protein expression.

TIGIT is expressed on human T cells and NK cells^18^,^19^. On CD8^+^ T cells, it is mainly expressed on intermediate/transitional and effector cells^23^. Here, we first set out to analyze the expression pattern of TIGIT in relation to its complementary co-stimulatory receptor CD226 and the inhibitory receptor PD-1 on different CD8^+^ T cell populations (gating strategy shown in Fig. 1a). The expression patterns of TIGIT, CD226 and PD-1 on naïve and memory CD8^+^ T cell populations (defined by the markers CD45RO, CCR7 and CD27) were visualized using viSNE maps in healthy (Supplementary Fig. S1) and HIV infected individuals (Fig. 1b–d). In all groups, TIGIT was expressed on all memory CD8^+^ T cell subsets but not on naïve CD8^+^ T cells, whereas PD-1 was more narrowly expressed on memory subpopulations within the central, effector, and transitional memory cells (Fig. 1d, and Supplementary Fig. S1). In contrast, CD226 had a broad range of expression, being highly expressed on all CD8^+^ T cell subsets including the naïve cells (Fig. 1c). The vast majority of cells expressing PD-1 were TIGIT^+^, however, not all TIGIT^+^ cells were PD-1^+^ demonstrating that TIGIT is more broadly expressed on CD8^+^ memory T cells than PD-1.

### Elevated levels of TIGIT^+^ CD8^+^ T cells despite early initiation of ART

**Figure 2:**
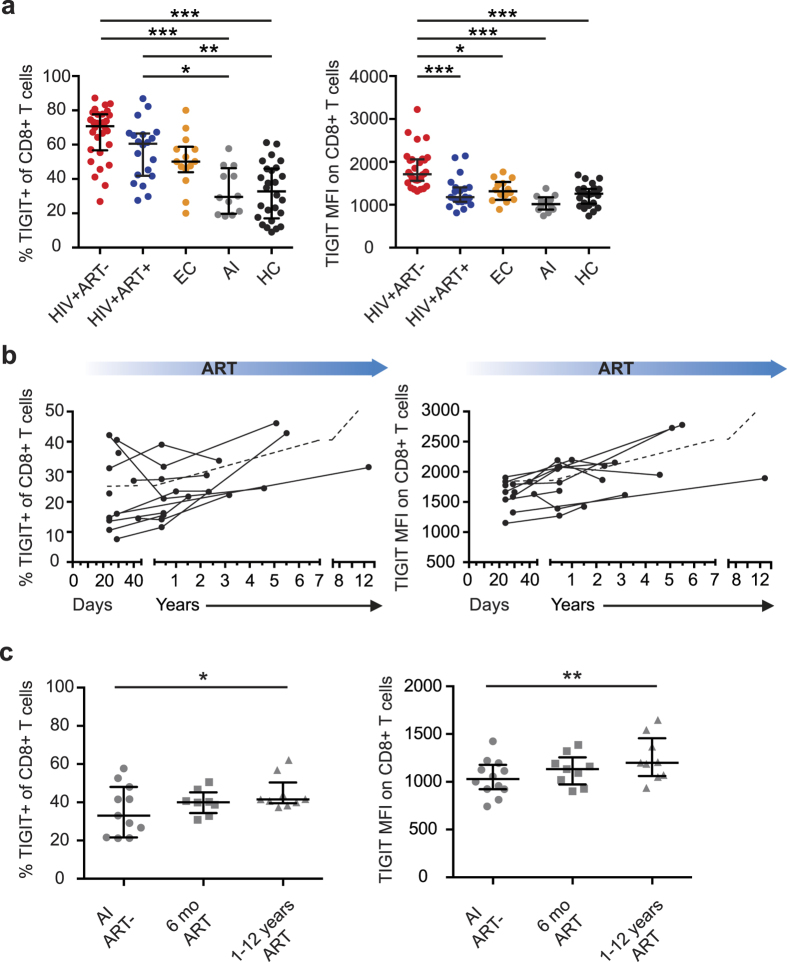
Expression of TIGIT on CD8^+^ T cells in HIV-positive subjects before and after ART (**a**) Frequency and MFI of TIGIT on CD8^+^ T cells in HIV+ treatment naïve (HIV+ART−, n = 30), long term treated (HIV+ART+, n = 20), elite controller (EC, n = 14), acutely infected (AI, n = 12) subjects and healthy controls (HC, n = 26). (**b**) Frequency and MFI of TIGIT on total CD8^+^ T cells over time in subjects followed from acute HIV infection (n = 12). Blue arrows indicate ART. (**c**) Frequency of TIGIT^+^ cells and TIGIT MFI on CD8^+^ T cells in subjects followed from acute HIV infection to chronic infection (n = 12). Circles represent acute HIV infection, squares represent the timepoint 6 months after ART initiation and triangles represent the timepoint 1–12 years after ART initiation. One-way ANOVA followed by Kruskall-Wallis test and Dunn’s multiple comparisons test were used to compare between ≥3 groups. The Wilcoxon matched-pairs signed rank test was used to compare paired samples. The Spearman non-parametric test was used for correlation analysis. *P < 0.05, **P < 0.01 and ***P < 0.001.

TIGIT expression was recently found to be increased on CD8^+^ T cells during chronic HIV-infection^23^. Here, we extended these studies and evaluated the longitudinal impact of ART on the expression levels of TIGIT. First, TIGIT expression was measured on total CD8^+^ T cells by multi-color flow cytometry in four groups of HIV-1-infected individuals; chronically infected treatment-naïve subjects, chronically infected successfully long-term treated subjects, elite controllers, and subjects with acute HIV-infection (Table 1, Fig. 2a). As compared to HIV-uninfected adults, the frequency of TIGIT^+^ CD8^+^ T cells was increased in chronic treatment-naïve and long-term treated subjects but not in elite controllers nor in individuals with acute infection. Similar patterns were observed when excluding the naïve CD8^+^ T cells from the analysis and when measuring the expression of PD-1, CD160 and 2B4 on total CD8^+^ T cells (Supplementary Fig. S2). However, treatment-naïve subjects had significantly higher TIGIT mean fluorescence intensity (MFI) compared to the long-term treated subjects, the other groups of HIV-positive subjects, and healthy controls (Fig. 2a and Supplementary Fig. S2).

We next investigated if early initiation of ART during acute HIV-infection would affect TIGIT expression levels. Longitudinal samples were collected (within median 26.5 days after the estimated infection date) from 10 study subjects initiated on ART during acute infection from two or three time-points up to 12 years (Table 1). Despite early initiation of ART, both the frequency of TIGIT^+^ cells and TIGIT MFI increased in the subsequent samples and was significantly higher in the samples obtained after one year on treatment (Fig. 2b and c). After one year, the frequency of TIGIT^+^ CD8^+^ T cells and TIGIT MFI on CD8^+^ T cells did not differ from long-term treated subjects in whom ART was initiated during chronic HIV-infection (Supplementary Fig. S2).

### TIGIT correlates with immunological markers of T cell pathology

**Figure 3:**
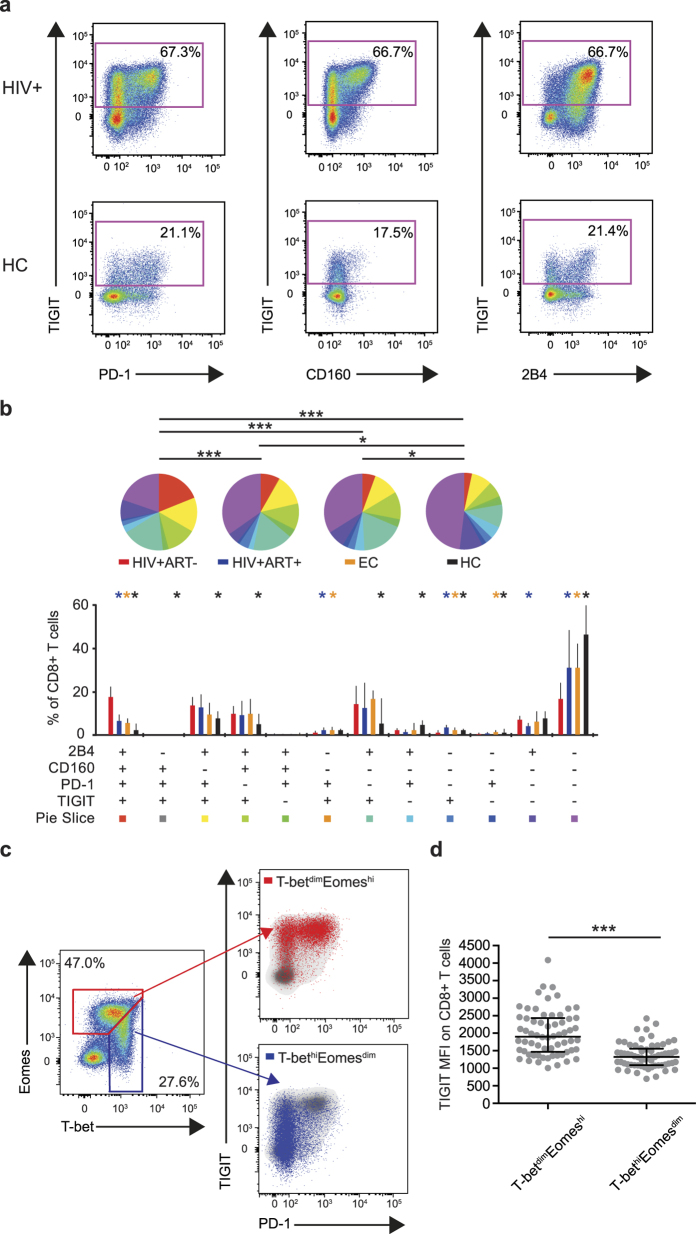
Expression of TIGIT, PD-1, CD160, 2B4 and T-box transcription factors on CD8^+^ T cells in HIV-positive subjects (**a**) Gating procedure for co-expression of TIGIT versus PD-1, CD160 and 2B4 on CD8^+^ T cells in a representative HIV+ subject (HIV+) and a healthy control (HC) subject. (**b**) SPICE analysis of combinations of TIGIT, PD-1, CD160 and 2B4 on total CD8^+^ T cells in HIV-positive treatment naïve (HIV+ART−, red, n = 30), long-term treated (HIV+ART−, blue, n = 20), elite controller (EC, orange, n = 14) subjects and healthy controls (HC, black, n = 26). (**c**) Expression of T-bet^dim^Eomes^hi^ (red) and T-bet^hi^Eomes^dim^ (blue) shown for total CD8^+^ T cells. Gating of T-bet and Eomes populations was performed according to previously used gating strategies^9^. (**d**) TIGIT expression on T-bet^dim^Eomes^hi^ and T-bet^hi^Eomes^dim^ populations on total CD8^+^ T cells in HIV+ART−, HIV+ART+ and EC subjects (n = 64). Permutation test was performed between the pie charts. Bar charts show median and IQR. The Wilcoxon matched-pairs signed rank test was used to compare paired samples. ***P < 0.001.

In addition to TIGIT, a number of other inhibitory receptors linked to immune dysfunction, such as PD-1, CD160 and 2B4^6^,^7^,^9^,^10^,^12^,^13^, are expressed on HIV-specific CD8^+^ T cells during the course of infection. However, since the link between TIGIT, PD-1, CD160 and 2B4 has not been investigated, we measured expression patterns of these markers on CD8^+^ T cells (Fig. 3a). Expression of TIGIT in combinations with PD-1, CD160 and 2B4 was significantly increased in the HIV-positive subject groups compared to healthy controls (Fig. 3b). Significant differences were seen between all groups except the chronically infected successfully long-term treated subjects and the elite controllers (Fig. 3b). The frequency of TIGIT^+^ cells was positively correlated with single- and triple expression of PD-1, CD160 and 2B4 (Supplementary Fig. S3) on CD8^+^ T cells.

T cell memory formation and functionality are controlled at the transcriptional level, and the T-box transcription factors T-bet and Eomes play an important role in these processes^15^,^16^. We, and others have recently shown that dysfunctional CD8^+^ T cells expressing high levels of PD-1, CD160 and 2B4^9^,^16^ have a specific T-bet^dim^Eomes^hi^ expression profile, while T-bet^hi^Eomes^dim^ cells define a less exhausted phenotype. Accordingly, we found that TIGIT MFI and the frequency of TIGIT^+^ cells were significantly higher in the T-bet^dim^Eomes^hi^ population compared to the T-bet^hi^Eomes^dim^ cells (Fig. 3c,d and Supplementary Fig. S4). Furthermore, the frequency of TIGIT^+^ CD8^+^ T cells correlated positively with the frequency of CD8^+^ T-bet^dim^Eomes^hi^ cells in chronically infected HIV-positive subjects (Supplementary Fig. S4). These data suggest that TIGIT is expressed on a highly exhausted population of CD8^+^ T cells.

Subsequently we investigated the relationship between TIGIT and clinical laboratory markers (viral load, CD4 count, CD4% and the CD4/CD8 ratio) and level of immune activation (CD38^+^HLA-DR^+^). Neither the frequency of TIGIT^+^ cells or the TIGIT MFI correlated with viral load in the chronically infected treatment-naïve subjects (excluding the elite controllers) (Supplementary Fig. S3). However, both the frequency of TIGIT^+^ cells and TIGIT MFI on CD8^+^ T cells were inversely correlated with CD4 count, the frequency of CD4^+^ T cells and the CD4/CD8 ratio (Supplementary Fig. S3). Furthermore, the frequency of CD38^+^HLA-DR^+^ cells was positively correlated to both the MFI and frequency of TIGIT^+^ cells (Supplementary Fig. S3). These results are in agreement with our previous findings in which the frequencies of CD4^+^ T cells and the CD4/CD8 ratio correlated strongly with PD-1 expression as well as immune activation^37^,^41^. These findings demonstrate that antiretroviral treatment initiated in chronic infection decreases TIGIT expression on TIGIT^+^ cells even though the frequency of TIGIT^+^ cells does not change significantly, which is in agreement with previous data for other inhibitory receptors^6^,^7^,^8^,^9^,^10^.

### Differential expression of TIGIT and CD226 on HIV-specific CD8^+^ T cells

**Figure 4:**
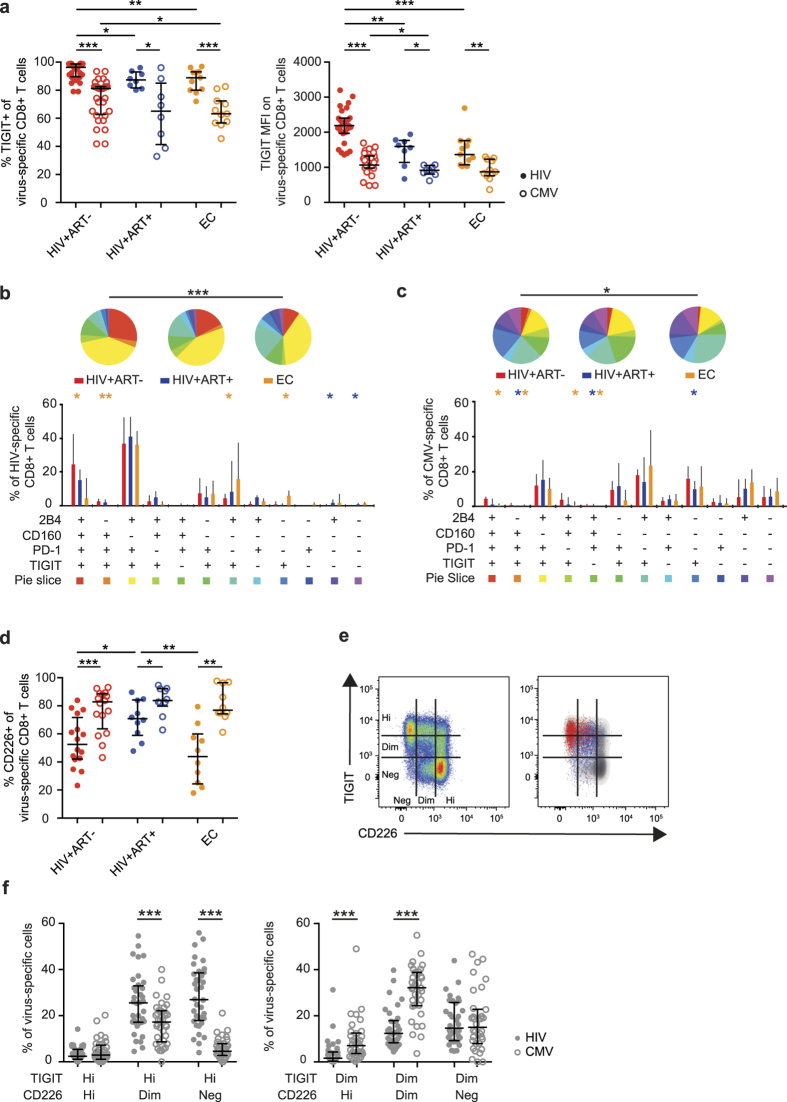
Linkage between TIGIT and CD226 expression on HIV-specific and CMV-specific CD8^+^ T cells. (**a**) Frequency and mean fluorescence intensity of TIGIT on HIV-specific (filled circles) and CMV-specific (open circles) CD8^+^ T cells in HIV-positive treatment-naive (HIV+ART−, red, n = 27), long-term treated (HIV+ART+, blue, n = 8) and elite controller (EC, orange, n = 11) subjects. (**b,c**) SPICE analysis of inhibitory receptor co-expression on HIV-specific CD8^+^ T cells (**b**) and CMV-specific CD8^+^ T cells (**c**) in HIV+ART− (n = 27), HIV+ART+ (n = 8) and EC (n = 11) subjects. (**d**) Frequency of CD226^+^ cells of HIV-specific (filled circles) and CMV-specific (open circles) CD8^+^ T cells in HIV+ART− (n = 15), HIV+ART+ (n = 10) and EC (n = 10) subjects. (**e**) Gating strategy for TIGIT hi/dim/neg and CD226 hi/dim/neg on total CD8^+^ T cells and an overlay of HIV-specific (red) and CMV-specific (blue) cells from a representative treatment naïve HIV infected subject. (**f**) Frequency of TIGIT^hi^ and TIGIT^dim^ HIV-specific (closed circles) and CMV-specific (open circles) CD8^+^ T cells that are CD226^hi^, CD226^dim^ and CD226^neg^ in chronically HIV infected subjects (n = 46). The Wilcoxon matched-pairs signed rank test was used to compare paired samples. The Mann-Whitney test was used to compare two groups. Permutation test was performed between the pie charts. Median and IQR are shown for all bars. *P < 0.05, **P < 0.01 and ***P < 0.001.

We next compared TIGIT expression on HIV-specific CD8^+^ T cells to CMV-specific cells that retain their effector capacity during chronic infection^42^,^43^. The vast majority of all HIV-specific CD8^+^ T cells expressed TIGIT, whereas CMV-specific cells displayed a significantly lower frequency of TIGIT^+^ cells and lower TIGIT MFI (Fig. 4a). The difference was most pronounced in the treatment naive and elite controller groups (Fig. 4a). The higher TIGIT MFI on HIV-specific cells was confirmed by tetramer staining identifying HLA-A2-restricted HIV-SL9-specific and CMV-NV9-specific CD8^+^ T cells in HIV+ subjects (n = 7) (Supplementary Fig. S4). Next, the co-expression pattern of TIGIT, PD-1, CD160 and 2B4 was measured on virus-specific CD8^+^ T cells. SPICE analysis revealed that treatment naïve subjects had a higher frequency of HIV-specific cells expressing all four inhibitory receptors compared to elite controllers (Fig. 4b). Three out of the four most common combinations contained TIGIT together with PD-1 (TIGIT^+^PD-1^+^CD160^+^2B4^+^, TIGIT^+^PD-1^+^CD160^−^2B4^+^, TIGIT^+^PD-1^+^CD160^−^2B4^−^) (Fig. 4b). In contrast, CMV-specific cells from the same subjects were more likely not to express inhibitory receptors on their surface and very few expressed all four inhibitory receptors (Fig. 4c).

Expression of TIGIT’s complementary stimulatory receptor CD226 is decreased on HIV- and melanoma-specific CD8^+^ T cells and cells with a high expression of PD-1^22^,^27^. However, the co-expression pattern of TIGIT and CD226 has not been investigated in HIV-infection. Here, we show that the frequency of CD226^+^ cells and CD226 MFI was lower on HIV-specific cells as compared to CMV-specific cells, especially in the treatment naïve and elite controller subjects (Fig. 4d and Supplementary Fig. S4). In the long-term treated subjects the frequency of CD226^+^ cells was partially restored, but remained lower than on CMV-specific cells from the same subjects (Fig. 4d). Next, the CD8^+^ T cells were divided into subpopulations (hi/dim/neg) based on intensity of TIGIT and CD226 expression (Fig. 4e). HIV-specific cells were predominantly TIGIT^hi^CD226^neg/dim^, whereas CMV-specific cells were TIGIT^dim^CD226^dim^ (Fig. 4f).

We next determined whether the TIGIT^+^ HIV-specific cells were activated (Supplementary Fig. S4). A positive correlation was found between the number of TIGIT^+^ cells and the number of highly activated (CD38^+^HLA-DR^+^) cells (Supplementary Fig. S4). These results show that the balance between CD226 and TIGIT expression is disrupted on HIV-specific cells and that HIV-specific cells expressing TIGIT are linked to a phenotype associated with severe dysfunction^9^.

### TIGIT^hi^ HIV-specific CD8^+^ T cells have a decreased functional capacity

**Figure 5:**
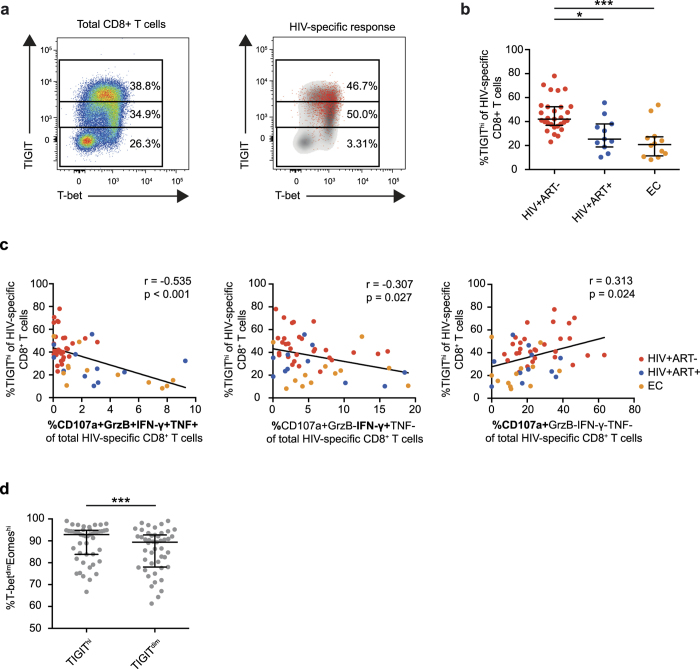
Functional capacity of HIV-specific CD8^+^TIGIT^hi^ cells (**a**) Gating procedure for TIGIT^hi^, TIGIT^dim^ and TIGIT^neg^ cells on CD8^+^ T cells in a representative treatment naïve HIV infected subject. (**b**) Frequency of TIGIT^hi^ cells of HIV-specific CD8^+^ T cells in HIV-positive treatment-naïve (HIV+ART−, n = 28), HIV+ long-term treated (HIV+ART+, n = 18) and elite controller (EC, n = 11) subjects. (**c**) Correlation between the frequency of TIGIT^hi^ cells and the frequency of polyfunctional CD8^+^ T cells or single expression of IFN-γ and CD107a on HIV-specific CD8^+^ T cells in HIV+ART− (red, n = 28), HIV+ART+ (blue, n = 18) and EC (orange, n = 11) subjects. (**d**) Frequency of T-bet^dim^Eomes^hi^ cells in the HIV-specific TIGIT^hi^ and TIGIT^dim^ population in HIV+ART− (n = 28), HIV+ART+ (n = 18) and EC (n = 11) subjects. One-way ANOVA followed by Kruskall-Wallis test and Dunn’s multiple comparisons test was used to compare between ≥3 groups. The Spearman non-parametric test was used for correlation analysis. The Wilcoxon matched-pairs signed rank test was used to compare paired samples. *P < 0.05 and ***P < 0.001.

As TIGIT MFI on HIV-specific cells was markedly higher in chronic untreated HIV-1 infection and discriminated HIV-specific from CMV-specific cells, we next investigated if the intensity of TIGIT expression on CD8^+^ T cells (Fig. 5a) was associated with their functional capacity. TIGIT^hi^ cells were more frequent in treatment-naïve subjects compared to long-term treated subjects and elite controllers (Fig. 5b and Supplementary Fig. S5). In contrast, only a small fraction of the CMV-specific cells were TIGIT^hi^ (Supplementary Fig. S5). Next, the functional capacity (expression of IFN-γ, TNF, granzyme B and CD107a) of TIGIT^hi^ HIV-specific CD8^+^ T cells was measured. The frequency of TIGIT^hi^ HIV-specific cells (measured by expression of IFN-γ) was inversely correlated with the frequency of polyfunctional CD107a^+^GrzB^+^IFN-γ^+^TNF^+^ cells as well as single expression of IFN-γ (Fig. 5c). A high frequency of polyfunctional CD8^+^ T cells was only detected in elite controllers and long-term treated subjects. On the contrary, single expression of CD107a correlated positively with the frequency of TIGIT^hi^ cells (Fig. 5c) and was primarily detected in the treatment-naïve subjects. In CMV-specific CD8^+^ T cells, the frequency of TIGIT^hi^ cells correlated inversely with the frequency of polyfunctional cells; however, no correlation was found for TIGIT^hi^ cells versus the frequency of cells expressing a single function (IFN-γ, TNF or CD107a) (Supplementary Fig. S5). This suggests that TIGIT^hi^ cells represent a dysfunctional population of HIV-specific CD8^+^ T cells that is most common in treatment naïve HIV-positive subjects. Furthermore we found that TIGIT^hi^ cells had a higher frequency of T-bet^dim^Eomes^hi^ cells as compared to TIGIT^dim^ cells (Fig. 5d). A similar observation has been made for PD-1^hi^ cells that are more likely to be Eomes^hi^ whereas PD-1^dim^ cells are T-bet^hi^ 16, suggesting that TIGIT^hi^ cells (similarly to PD-1^hi^ cells) represent a more transcriptionally exhausted phenotype.

### Expression of the TIGIT ligand PVR is increased on CD4^+^ T cells from HIV-infected lymph nodes

**Figure 6:**
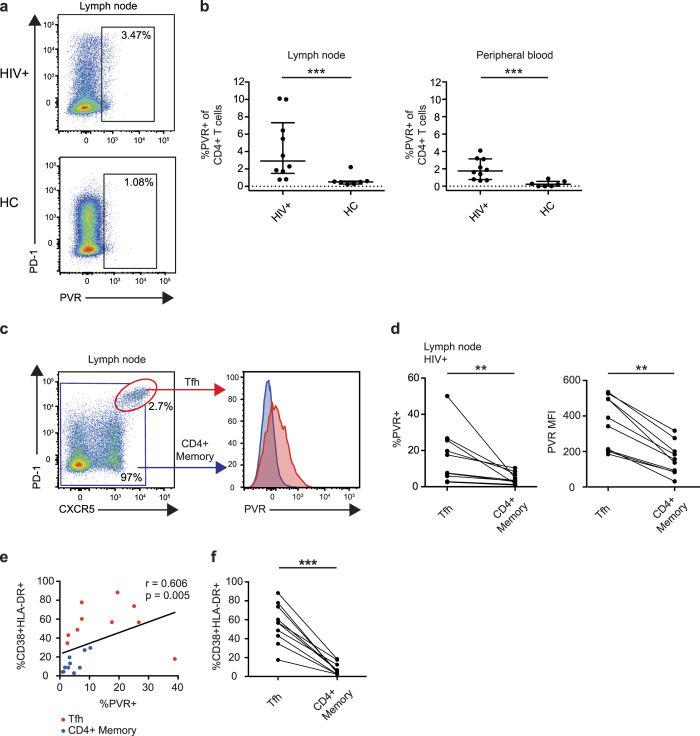
PVR expression on CD4^+^ T cells in peripheral blood and lymph node. (**a**) Gating strategy for PVR^+^ cells of total CD4^+^ T cells from a representative HIV+ and healthy control (HC) subject. (**b**) Frequency of PVR^+^ cells of CD4^+^ T cells in HIV+ (n = 10) and HC (n = 7) subjects in lymph node and peripheral blood. (**c**) Gating strategy for PVR expression on T follicular helper (Tfh) cells and histogram showing PVR expression of Tfh cells (red) and memory CD4^+^ T cells (blue). (**d**) Frequency of PVR^+^ cells of Tfh cells versus memory CD4^+^ T cells in lymph nodes of HIV infected subjects (n = 10). (**e**) Correlation analysis of the frequency of CD38^+^HLA-DR^+^ cells versus frequency of PVR expression on Tfh cells (red) and CD4^+^ memory T cells (blue). (**f**) Comparison between the frequency of CD38^+^HLA-DR^+^ cells of Tfh versus CD4^+^ memory cells in the lymph nodes from HIV infected subjects (n = 10). The Mann-Whitney test was used for comparisons between two groups. The Wilcoxon matched-pairs signed rank test was used to compare paired samples. The Spearman non-parametric test was used for correlation analysis. **P < 0.01 and ***P < 0.001.

To exert its inhibitory function, TIGIT must bind to its ligand PVR on antigen presenting cells. However, the expression of PVR in HIV-positive subjects has not been investigated *ex vivo*. For that purpose, we measured PVR expression on CD4^+^ T cells from peripheral blood and lymph nodes in treatment naïve HIV-positive subjects (n = 10) and healthy controls (n = 8) (Fig. 6a and b). Expression of PVR on CD4^+^ T cells was significantly higher in both lymph node and peripheral blood in HIV-positive individuals, compared to healthy controls (Fig. 6b). Further, since numbers of T follicular helper cells (Tfh) cells are expanded and represent a major reservoir of HIV^33^,^34^, we next determined PVR expression on these cells in HIV-positive subjects. Tfh cells were defined by an expression profile of CD4^+^PD-1^hi^CXCR5^+^ (Fig. 6c) and were found mainly in the lymph nodes. In HIV-infected subjects, Tfh cells had a higher frequency of PVR^+^ cells and a higher PVR MFI compared to memory CD4^+^ T cells in the same subjects (Fig. 6d). The increased expression of PVR could be a result of an increased activation of CD4^+^ T cells. Indeed, the frequency of CD4^+^ T cell activation and PVR expression correlated positively on Tfh and CD4^+^ memory T cells (Fig. 6e) and Tfh cells displayed a more activated phenotype as compared to CD4^+^ memory cells (Fig. 6f). These data demonstrate that PVR is increased on CD4^+^ T cells and is highly elevated on Tfh cells in HIV-infected subjects. Although PVRL2 (CD112) was also measured in these samples, no significant differences were found (data not shown).

## Discussion

HIV-1 infection is characterized by severe perturbation of the cellular immune system, a process referred to as immune exhaustion. This process has previously been linked to the co-expression of several inhibitory receptors, including PD-1, CD160, 2B4, TIM-3 and LAG-3 on CD8^+^ T cells^5^,^9^,^10^, poor functionality^44^,^45^ and an altered transcriptional profile^9^. A recently described inhibitory receptor, TIGIT, known to inhibit the function of T cells and NK cells^17^,^18^,^19^, is expressed on the majority of HIV-specific CD8^+^ T cells^23^ and tumor-infiltrating lymphocytes^21^,^22^. Here, in a set of longitudinal samples, we report an increased TIGIT expression over time despite early intervention with ART during acute HIV infection. The expression of TIGIT was closely linked to expression of the inhibitory receptors PD-1, CD160 and 2B4 and to a T-bet^dim^Eomes^hi^ transcriptional profile. Additionally, TIGIT^hi^ HIV-specific CD8^+^ T cells were predominantly CD226^dim/neg^ and associated with a poor functional capacity. Furthermore, we demonstrate that expression of the TIGIT/CD226 ligand, PVR was increased on Tfh cells in HIV-positive subjects, which represent a major reservoir for HIV-1, adding to the number of obstacles one must overcome to clear HIV-1 infected cells by immune-based curative strategies.

First, in agreement with recent report by Chew *et al*.^23^, we show an increased expression of TIGIT on bulk and memory CD8^+^ T cells in HIV-positive subjects. We further extended these recent data by demonstrating in longitudinal samples from subjects followed from acute to chronic HIV-infection, that ART did not prevent the increase of TIGIT expression, nor did it significantly decrease the frequency of TIGIT^+^ cells in subjects treated from chronic infection. A similar pattern was previously shown for PD-1 expression, where a decrease in viral load after treatment was more strongly related to the decreased MFI of PD-1 than the frequency of PD-1^+^ cells^6^,^8^,^9^,^10^. Furthermore, this process might be influenced by the establishment of the viral reservoir within lymphoid tissues, i.e. infection of the Tfh cells^34^ and the suggested ongoing viral replication despite successful ART^46^. However, it remains to be identified whether the elevated TIGIT expression levels in individuals treated from acute infection might be associated with persistent RNA replication within the viral reservoirs.

We next demonstrated that the inhibitory receptors PD-1, CD160 and 2B4, expressed on exhausted CD8^+^ T cells in HIV-infection^9^,^10^, were linked to TIGIT expression on bulk and HIV-specific CD8^+^ T cells. Our data further show that a high level of viral replication is linked to the markedly higher frequency of CD8^+^ T cells expressing TIGIT, PD-1, CD160 and 2B4, seen in untreated subjects as compared to subjects with undetectable viral load (long-term treated subjects and elite controllers). Importantly, long-term ART did not restore the levels of inhibitory receptor expression to levels seen in healthy subjects, as previously shown for PD-1, CD160 and 2B4^9^, suggesting that a decrease in viral load alone does not fully restore CD8^+^ T cells in HIV-infection.

Previous studies on PD-1^+^ cells have shown different functional properties and differential expression of T-bet and Eomes depending on the intensity of PD-1 expression^16^. Additionally, TIGIT can repress T-bet expression in human CD4^+^ T cells^47^ and our group recently linked cells with a T-bet^dim^Eomes^hi^ transcriptional profile to a population of highly exhausted CD8^+^ T cells in chronic HIV infection^9^. The results show that exhausted T cells during HIV-infection lose their expression of T-bet, which is linked to the functional impairment of the CD8^+^ T cells and thus an inability to clear infected cells. Here, we accordingly link the T-bet^dim^Eomes^hi^ cells phenotype to an increased expression of TIGIT in HIV-infection. However it remains to be determined whether T-bet and Eomes are able to directly regulate TIGIT expression on CD8^+^ T cells.

TIGIT expression is increased on HIV-specific CD8^+^ T cells and CD8^+^ tumor-infiltrating lymphocytes^21^,^22^,^23^, and a decreased CD226 expression on PD-1^+^CD8^+^ T cells is associated with an increase in viral load in chronically HIV infected subjects^27^. Activation of CD8^+^ T cells by non-professional antigen-presenting cells, such as CD4^+^ T cells, requires expression of CD226^48^, and is likely of importance for HIV-specific CD8^+^ T cell recognition of infected CD4^+^ T cells. We here demonstrate that HIV-specific TIGIT^hi^ CD8^+^ T cells were mainly CD226^dim/neg^, likely due to the ability of TIGIT to disrupt CD226 dimerization^21^. These data suggest that the role of CD226 as a co-stimulatory molecule is limited in HIV-infection. Interestingly, co-blockade of TIGIT and PD-1 on CD8^+^ T cells was recently shown to be less effective on tumor infiltrating lymphocytes as compared to circulating melanoma-specific CD8^+^ T cells in patients with metastatic melanoma. This might be related to the expression of CD226, which is expressed by most tumor-specific cells in the periphery but downregulated on tumor-infiltrating lymphocytes^22^. Therefore, the effect of TIGIT blockade is likely to be related to expression of CD226. The modest effect on HIV-specific CD8^+^ T cell function after co-blockade of TIGIT and PD- seen by Chew *et al*.^23^ might therefore be explained by the loss of CD226, however this remains to be determined. The expression pattern of TIGIT^hi^CD226^neg/dim^ on HIV-specific cells shown here likely represents a way for the immune system to balance the antiviral response in order to limit immune damage by increasing inhibition and decreasing activation. However, in terms of restoring CD8^+^ T cells for immune based cure strategies^49^, this process becomes troublesome, as the CD8^+^ T cell responses are dampened.

Additionally the frequency of HIV-specific TIGIT^hi^ CD8^+^ T cells was negatively associated with polyfunctionality measured as co-expression of IFN-γ, TNF, granzyme B and CD107a, as well as single expression of IFN-γ. Interestingly, the frequency of HIV-specific TIGIT^hi^ CD8^+^ T cells was positively correlated with the number of CD107a single positive cells. In our previous studies, single expression of CD107a was linked to a highly exhausted phenotype^9^. Thus, the TIGIT^hi^ CD8^+^ T cells have the capacity to degranulate, but most degranulating cells do not express granzyme B, suggesting that the cells may not be able to eliminate infected cells. However this remains to be determined.

TIGIT induced inhibition of degranulation requires TIGIT binding to its ligand PVR on target cells^20^ (*e.g.* HIV-infected CD4^+^ T cells). We found an increased expression of PVR on CD4^+^ T cells from lymph nodes and peripheral blood of HIV-infected individuals. Interestingly, the highest levels of PVR expression on CD4^+^ T cells was found on Tfh cells, providing evidence that PVR expression could be increased on HIV infected CD4^+^ T cells. Data from HIV- and SIV-infection show that HIV- and SIV-specific cells do not generally enter the B cell follicles where Tfh cells reside^50^,^51^. Our data suggest, that even if CD8^+^ T cells were to reach the B cell follicles, their cytolytic function would likely be limited due to the high expression of TIGIT and PVR and the loss of CD226 expression. This creates a barrier for HIV-specific CD8^+^ T cells to overcome in order to eliminate the HIV reservoir.

In summary, our findings show an increased expression of TIGIT on bulk and HIV-specific CD8^+^ T cells during HIV-infection coupled to increased expression of the inhibitory markers PD-1, CD160 and 2B4, a T-bet^dim^Eomes^hi^ transcriptional profile and loss of functional capacity. Importantly, early initiation of ART did not prevent a steady longitudinal increased expression of TIGIT. In addition, expression of the TIGIT’s complementary co-stimulatory molecule CD226 was decreased on HIV-specific CD8^+^ T cells and expression of their shared ligand PVR was increased on CD4^+^ T cells, specifically on Tfh cells. Our results demonstrate a perturbation of the TIGIT/CD226/PVR axis linked to multiparametric T cell pathology in HIV infection despite ART. This outcome suggests that future immune-based curative strategies will need to reverse the imbalance of the TIGIT/CD226/PVR axis caused by HIV.

## Additional Information

**How to cite this article**: Tauriainen, J. *et al*. Perturbed CD8^+^ T cell TIGIT/CD226/PVR axis despite early initiation of antiretroviral treatment in HIV infected individuals. *Sci. Rep.* **7**, 40354; doi: 10.1038/srep40354 (2017).

**Publisher's note:** Springer Nature remains neutral with regard to jurisdictional claims in published maps and institutional affiliations.

## Supplementary Material

Supplementary Figures

## Figures and Tables

**Table 1:** Patient characteristics.

Group	HIV+ART−	HIV+ART+	EC	AI	HC
No of subjects	30	20	14	12	31
Age	40 (33–47)	48 (43–55)	48 (41–54)	34 (28–43)	33 (31–40)
Gender	57% M	70% M	80% M	100% M	68% M
CD4 count (cells/mm^3^)	419 (302–552)	700 (590–940)	1110 (719–1399)	575 (343–818)	N.A
CD4%	21 (17–27)	36 (30–42)	43 (32–50)	N.A	N.A
CD4/CD8	0.38 (0.24–0.66)	0.92 (0.81–1.34)	1.26 (0.93–1.75)	0.74 (0.26–1.05)	N.A
VL (copies/mL)	31,000 (7,390–188,750)	<50	<50	500,000 (100,579–1,530,000)	N.A
Time after estimated infection date	3 (1–7) years	16 (12–20) years	N.A	27 (24–30) days	N.A
Years on treatment	Naïve	14 (10–17)	Naïve	Naïve	N.A

Median and IQR are shown for all values.

HIV+ART−: HIV-infected treatment naïve subjects; HIV+ART+: HIV-infected subjects on long-term (>6 yrs) treatment; EC: Elite controllers; AI: Subjects with acute HIV-infection; HC: Healthy controls; M: Male; VL: Viral load; N.A: Not Available.
