# Supplementary material for: Perturbed CD8+ T cell TIGIT/CD226/PVR axis despite early initiation of antiretroviral treatment in HIV infected individuals
Source: Sci Rep. 2017 Jan 13;7:40354. doi: 10.1038/srep40354 (PMC5233961; doi:10.1038/srep40354)
Supplement: Supplementary Figures [file srep40354-s1.pdf]

Perturbed CD8$^+$ T cell TIGIT/CD226/PVR axis despite early initiation of antiretroviral treatment in HIV infected individuals

Authors

Johanna Tauriainen[1], Lydia Scharf [1], Juliet Frederiksen[2], Ali Naji[3], Hans-Gustaf Ljunggren[4], Anders Sönnerborg[1,5], Ole Lund[2], Gustavo Reyes-Terán[6], Frederick M Hecht[7], Steven G Deeks[7], Michael R Betts[8], Marcus Buggert[4,8], Annika C Karlsson[1*]

Affiliations

[1]Division of Clinical Microbiology, Department of Laboratory Medicine, Karolinska Institutet, Karolinska University Hospital Huddinge, Stockholm, Sweden. [2]Center for Biological Sequence Analysis, Department of Systems Biology, Technical University of Denmark, Lyngby, Denmark. [3]Division of Transplantation, Department of Surgery, Perelman School of Medicine, University of Pennsylvania, Philadelphia, PA, United States of America. [4]Center for Infectious Medicine, Department of Medicine, Karolinska Institutet, Karolinska University Hospital, Stockholm, Sweden. [5]Unit of Infectious Diseases, Department of Medicine Huddinge, Karolinska Institutet, Karolinska University Hospital Huddinge, Stockholm, Sweden [6]Centre for Infectious Diseases Research, National Institute of Respiratory Diseases, Mexico City, Mexico [7]Department of Medicine, University of California, San Francisco Positive Health Program, San Francisco General Hospital, San Francisco, CA, United States of America. [8]Department of Microbiology, Perelman School of Medicine, University of Pennsylvania, Philadelphia, PA, United States of America.

* Correspondence to annika.karlsson@ki.se

Supplementary figures

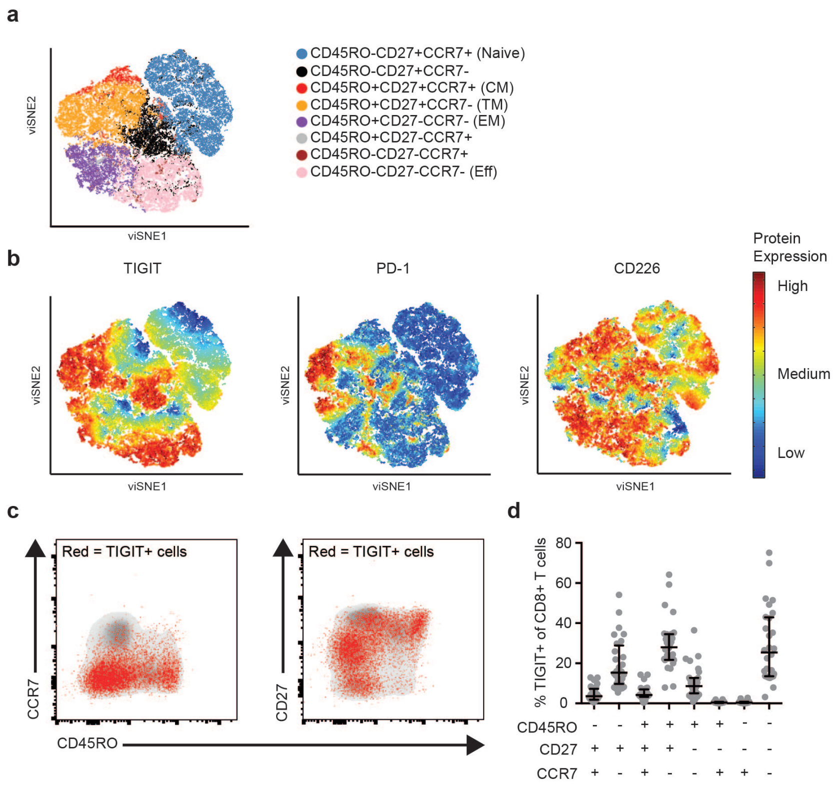

Supplementary figure 1. Expression of TIGIT, CD226 and PD-1 on CD8+ T cells in healthy control subjects. a) viSNE map of naïve/memory phenotype distribution (based on CD45RO, CCR7 and CD27) in healthy (n=26) subjects (b) viSNE maps of expression of TIGIT, CD226 and PD-1 on naïve/memory phenotypes corresponding to S1a. Colors depict intensity of protein expression. (c) Flow plots confirming the expression pattern of TIGIT on $CD8^+$ T cell memory subtypes (density plot: $CD8^+$ T cells; red: $TIGIT^+$ cells). (d) Frequency of $TIGIT^+$ cells in $CD8^+$ T cell naïve/memory populations in the healthy subjects, based on expression of CD45RO, CCR7 and CD27.

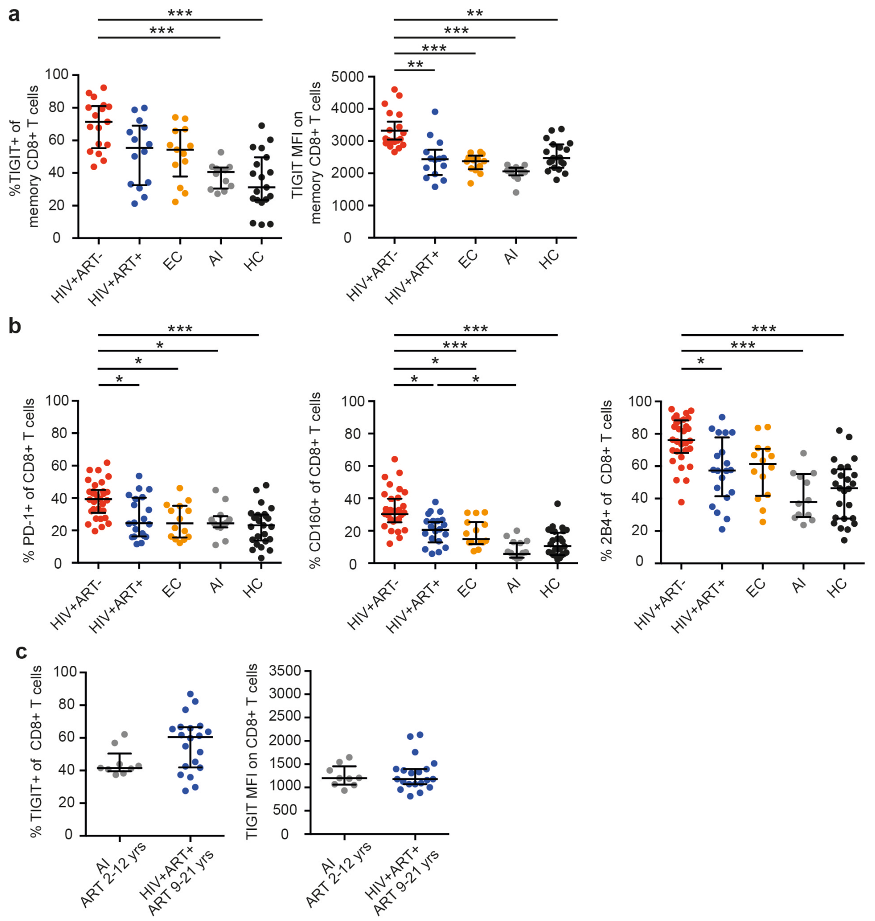

Supplementary figure 2. Expression of TIGIT, PD-1 and 2B4 on CD8+ T cells in HIV-positive subjects before and after ART. (a) Frequency of TIGIT^{+} cells and TIGIT MFI on memory CD8^{+} T cells in HIV+ treatment naïve subjects (HIV+ART-, red, n=17), long-term treated subjects (HIV+ART+, blue, n=13), elite controllers (EC, orange, n=13), subjects with acute infection (AI, grey, n=11) and healthy controls (HC, black, n=19). (b) Frequency of PD-1^{+}, CD160^{+} and 2B4^{+} cells of total CD8^{+} T cells in HIV+ART- (n=30), HIV+ART+ (n=20), EC (n=14), AI (n=12) and HC (n=26) subjects. (c) Frequency of TIGIT^{+} cells and TIGIT MFI on CD8^{+} T cells in HIV+ subjects treated from acute infection for more than a year (AI, ART 2-12 yrs, grey, n=9), and chronically infected long-term treated subjects (HIV+ART+, ART 9-21 yrs, blue, n=20). One-way ANOVA followed by Kruskall-Wallis test and Dunn's multiple comparisons test were used to compare between ≥ 3 groups. *P < 0.05, **P < 0.01 and ***P < 0.001

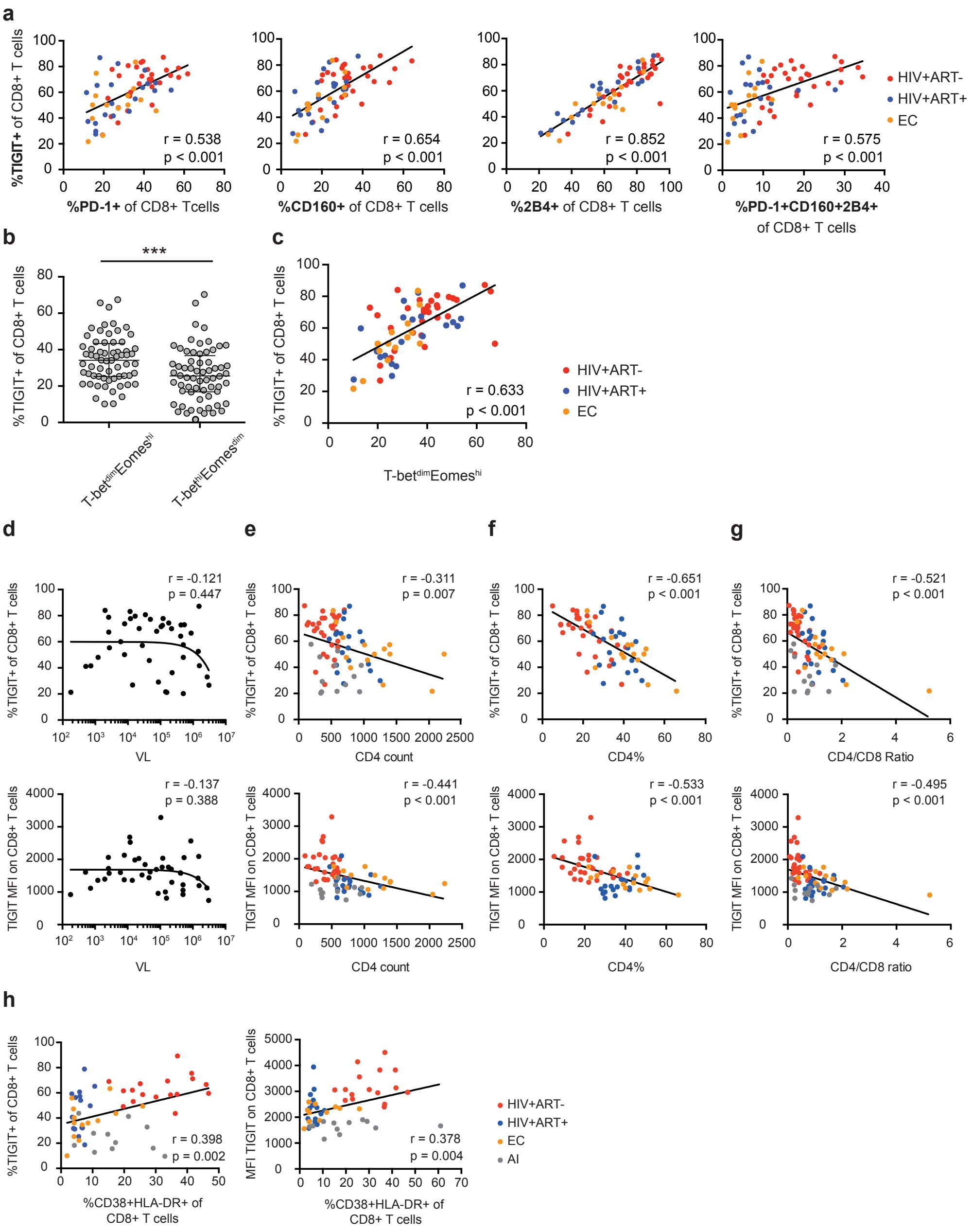

Supplementary figure 3. TIGIT expression in association with PD-1, CD160 and 2B4 expression and T-box transcription factors on CD8$^+$ T cells in HIV-positive subjects. (a) Correlation analysis of single and triple-expression of the inhibitory receptors PD-1, CD160 and 2B4 versus TIGIT on total CD8$^+$ T cells in HIV-positive treatment naïve (HIV+ART-, red, n=30), long-term treated (HIV+ART+, blue, n=20) and elite controller (EC, orange, n=14) subjects. (b) Frequency of T-bet^{dim}Eomes^{hi} and T-bet^{hi}Eomes^{dim} cells of TIGIT$^+$CD8$^+$ T cells in HIV+ART-, HIV+ART+ and EC subjects (n=64). (c) Correlation analysis between the frequency of TIGIT$^+$ CD8$^+$ T cells and T-bet^{dim}Eomes^{hi} CD8$^+$ T cells in HIV+ART- (n=30), HIV+ART+ (n=20) and EC (n=14) subjects. (d) Correlation analysis of the frequency of TIGIT$^+$ cells and TIGIT MFI on total CD8$^+$ T cells versus viral load (VL) in treatment naïve HIV infected subjects (HIV+ART-, n=20), (e) CD4 count in HIV+ART- (red, n=30), long-

term treated HIV infected (HIV+ART+, blue, n=20), elite controller (EC, orange, n=14) and acutely infected (AI, grey, n=12) subjects. (f) Frequency of $CD4^+$ T cells in HIV+ART-, HIV+ART+ and EC subjects and (g) CD4/CD8 ratio in HIV+ART-, HIV+ART+, EC and AI subjects. (h) Correlation analysis of the frequency of $TIGIT^+CD8^+$ T cells and activated ($CD38^+HLA\text{-}DR^+$) $CD8^+$ T cells in HIV+ART-, HIV+ART+, EC and AI subjects. Permutation test was performed between the pie charts. Bar charts show median and IQR. The Wilcoxon matched-pairs signed rank test was used to compare paired samples. The Spearman non-parametric test was used for correlation analysis. $^*P < 0.05$, and $^{***}P < 0.001$.

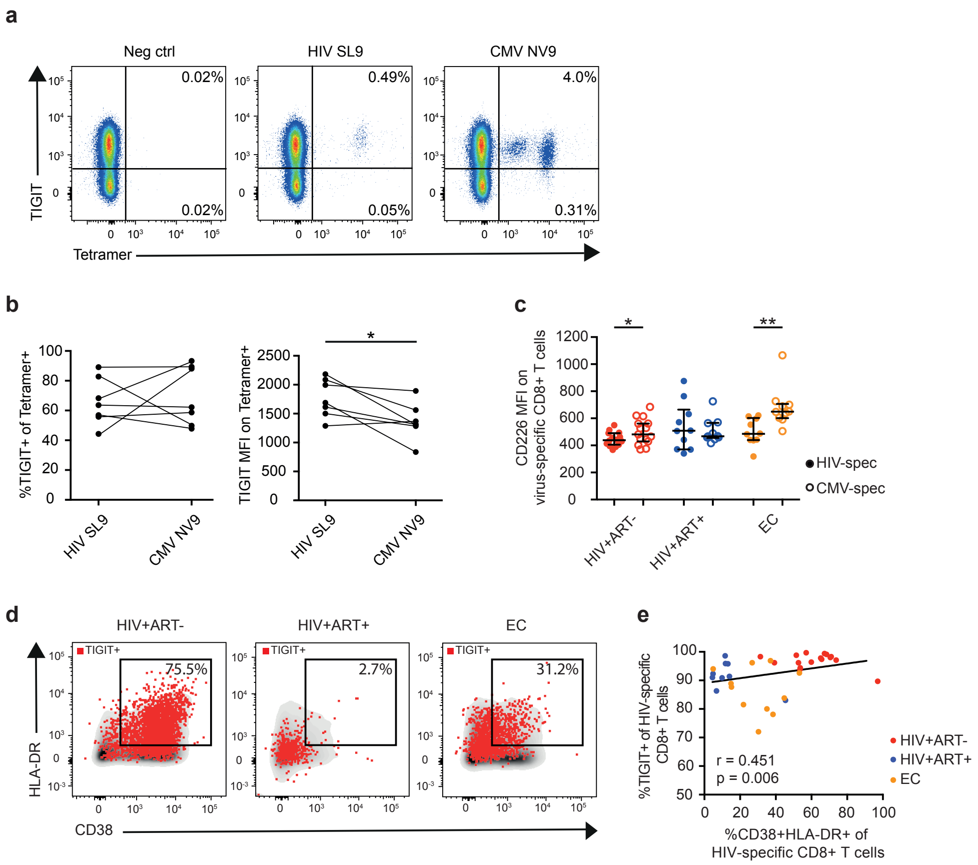

Supplementary figure 4. Expression of TIGIT and CD226 on HIV-specific and CMV-specific CD8+ T cells. (a) Gating procedure for tetramer staining with HIV SL9 and CMV NV9 on total CD8$^+$ T cells in a representative HIV+ subject. (b) TIGIT MFI on CD8$^+$ HIV SL9$^+$ and CD8$^+$ CMV NV9$^+$ cells in HIV+ subjects (n=7). (c) CD226 MFI on HIV-specific (filled circles) and CMV-specific (open circles) CD8$^+$ T cells in HIV+ treatment naïve (HIV+ART-, red, n=16) long-term treated (HIV+ART+, blue, n=13) and elite controller (EC, orange, n=11) subjects. (d) Gating strategy for activated HIV-specific TIGIT$^+$CD8$^+$ T cells in a representative HIV+ART-, HIV+ART+ and EC subject. (e) Correlation analysis between the frequency of TIGIT$^+$ and CD38$^+$HLA-DR$^+$ CD8$^+$ T cells in HIV+ART- (n=16), HIV+ART+ (n=13) and EC (n=11) subjects. The Wilcoxon matched-pairs signed rank test was used to compare paired samples. The Mann-Whitney test was used for comparisons between two groups. The Spearman non-parametric test was used for correlation analysis. *$P < 0.05$ and **$P < 0.01$

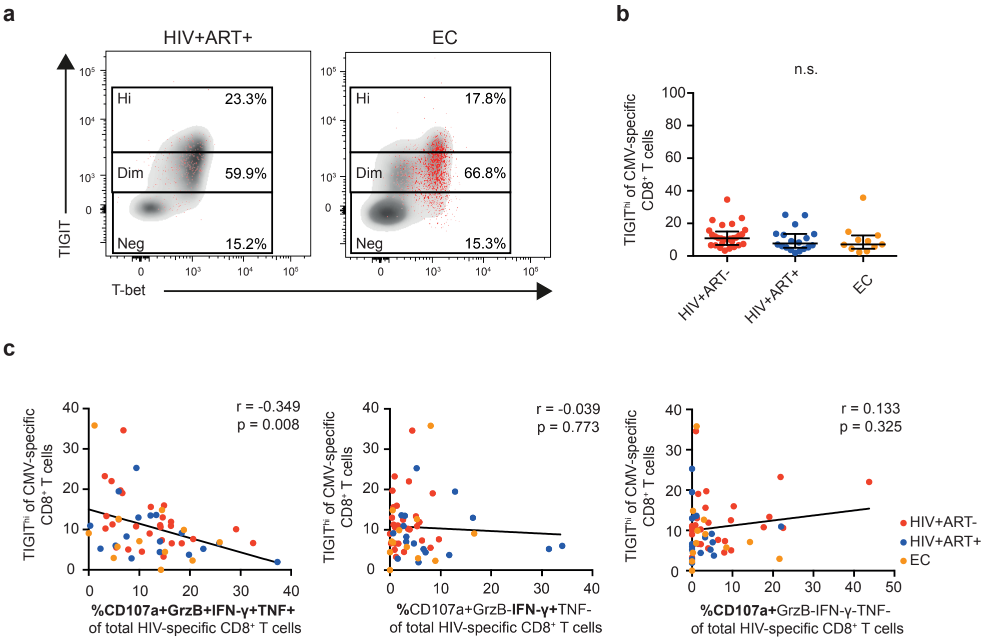

Supplementary figure 5. Functional capacity of antigen-specific CD8^{+}TIGIT^{hi} cells (a) Gating strategy for TIGIT^{hi}, TIGIT^{dim} and TIGIT^{neg} cells for total CD8^{+} T cells (density plot) and HIV-specific CD8^{+} T cells (red) in a representative long-term treated HIV+ subject (HIV+ART+) and elite controller (EC). (b) Frequency of TIGIT^{hi} cells of CMV-specific CD8^{+} T cells in HIV-positive treatment naive (HIV+ART-, red, n=28), long-term treated (HIV+ART-, blue, n=18) and elite controller (EC, orange, n=11) subjects. (c) Correlation analysis of TIGIT^{hi} CMV-specific cells versus CD107a^{+}GrzB^{+}IFN-γ $^{+}$TNF^{+} cells and IFN-γ and CD107a single positive cells in HIV+ART- (n=28), HIV+ART+ (n=18) and EC (n=11) subjects. One-way ANOVA followed by Kruskall-Wallis test and Dunn's multiple comparisons test was used to compare between ≥ 3 groups. The Spearman non-parametric test was used for correlation analysis. N.s. = no significance.
